# Abstracts and Selections

**Published:** 1911-01

**Authors:** 


### Abstracts and Selections.

"Call him not old, whose visionary brain
Holds o'er the past its undivided reign.
For him in vain the envious seasons roll
Who bears eternal summer in his soul.
If yet the minstrel's song, the poet's lay,
Spring with her birds, or children with their play,
Or maiden's smile, or heavenly dreams of art
Stir the life-drops creeping round his heart,—
Turn to the record where his years are told,—
Count his gray hairs, they can not make him old!"

—*Holmes.*

I WANT the boys and girls to build up character in themselves and in the community, to give to the country just so many men and women who will be incapable of meanness or dishonesty, who will look upon life as a sacred trust, given to them for honorable service to their fellow men and women. I would have them feel that, whether rich or poor, they are bound to be of use in their day and generation, and to be mindful of the scripture saying that "no man liveth unto himself." We all have our part to do in keeping up the character and credit of our country. For her sake we should study to become good and useful citizens.—*Pilgrim Teacher.*

